# The Dangers of Gelatin Injections

**Published:** 1904-05

**Authors:** 


### Reviews of Dental Literature.

The Dangers of Gelatin Injections.—Since gelatin injections were first recommended for the control of severe hemorrhages in various organs, and for the treatment of aortic aneurism, they have been used quite extensively. Much has been said in favor of this method, but several deaths have resulted. The fatal results were due to such complications as septic thrombosis, phlegmons, malignant œdema, and particularly to tetanus.

Several communications have recently appeared which again strongly emphasize the dangers connected with the subcutaneous injection of gelatin. Chauffard reported a fatal case of tetanus following a gelatin injection, and states that seventeen similar cases have previously been placed on record. Dieulafoy a few weeks later reports another instance of fatal tetanus developing after a gelatin injection given to control a severe tubercular hæmoptysis, and he adds four more cases not included in Chauffard's statistics, bringing the total number of such deaths to twenty-three.

Quite an elaborate study on the dangers and the therapeutic value of gelatin injection has just been published by Doerfler, who has twice lost patients from tetanus following subcutaneous application of gelatin. In one instance the method was resorted to to control a severe postpartum hemorrhage, in the other case to stop a tubercular hæmoptysis.

It is now well known that tetanus following gelatin injections is almost universally due to the introduction of tetanus spores with the gelatin in which they were contained. Levy and Bruns obtained bacilli from eight out of thirteen samples of gelatin examined. It was formerly believed that the spores of the tetanus bacillus are killed by an exposure during eight minutes to streaming steam at 100° C., but the authors quoted have shown that even after thirty minutes' exposure some tetanus spores survive, and only after thirty-three minutes were all samples of infected gelatin so exposed found absolutely free from tetanus germs. According to Forster and Brehmer larger masses of gelatin must be exposed forty minutes to a temperature of 100° to 120° C., and this after a preliminary warming, before all tetanus spores are killed. Many of those who have used gelatin injections have not been aware that it is necessary to so extend the period of sterilization.

In quite a number of fatal cases of tetanus following gelatin injections it was subsequently demonstrated that the gelatin used contained tetanus bacilli or spores. In other cases the sterilized gelatin left was found free from living tetanus spores. We understand now that the sterilization was insufficient, and while it may have made one part of the gelatin free from tetanus spores, it left them alive in other portions.

Doerfler states that prolonged and energetic sterilization does not at all interfere with the therapeutic value of gelatin, and that if indeed sterile it is absolutely void of any danger. He concedes that the great hopes placed in gelatin injections in the treatment of aortic aneurism have not been realized, but that it has done excellent service in tubercular hæmoptysis, or pulmonary hemorrhage from other causes, in intestinal, renal, vesical, and uterine hemorrhages, in hæmophilia, and in melena neonatorum.—*Journal of the American Medical Association,* July 4, 1903.

